# Carbonyl Emissions and Heating Temperatures across 75 Nominally Identical Electronic Nicotine Delivery System Products: Do Manufacturing Variations Drive Pulmonary Toxicant Exposure?

**DOI:** 10.1021/acs.chemrestox.2c00391

**Published:** 2023-02-16

**Authors:** Soha Talih, Rola Salman, Nareg Karaoghlanian, Ahmad El-Hellani, Alan Shihadeh

**Affiliations:** † Mechanical Engineering Department, Maroun Semaan Faculty of Engineering and Architecture, American University of Beirut, Bliss Street, P.O. Box 11-0236, Beirut, Lebanon; ‡ Center for the Study of Tobacco Products, Virginia Commonwealth University, 821 West Franklin Street, Richmond, Virginia 23284, United States; § Environmental Health Sciences, College of Public Health, The Ohio State University, 1841 Neil Ave., Columbus, Ohio 43210, United States

## Abstract

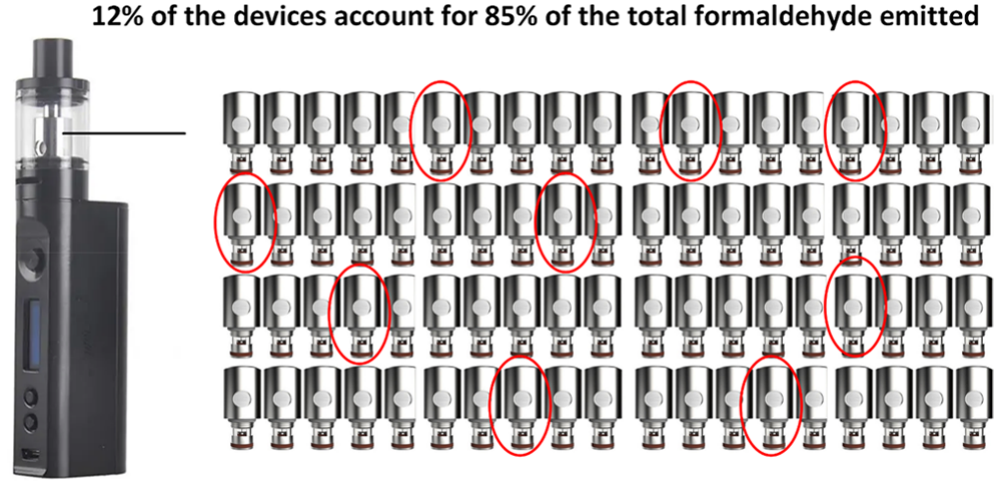

Studies of factors that impact electronic nicotine delivery systems (ENDSs) carbonyl compound (CC) emissions have been hampered by wide within-condition variability. In this study, we examined whether this variability may be related to heating coil temperature variations stemming from manufacturing differences. We determined the mean peak temperature rise (Δ*T*_max_) and CC emissions from 75 Subox ENDSs powered at 30 W. We found that Δ*T*_max_ and CC emissions varied widely, with greater Δ*T*_max_ resulting in exponentially higher CC emissions. Also, 12% of atomizers accounted for 85% of total formaldehyde emissions. These findings suggest that major reductions in toxicant exposure might be achieved through regulations focusing on limiting coil temperature.

Electronic nicotine delivery systems (ENDSs) deliver nicotine by heating and aerosolizing a solution that contains propylene glycol (PG), vegetable glycerin (VG), nicotine, and other additives. When a puff is drawn, electrical power flows through a heating coil that heats and vaporizes the solution. In the process, carbonyl compounds (CCs) form by thermal decomposition of PG and VG.^1^ CCs have drawn significant attention in the scientific literature because several of these compounds are highly toxic or carcinogenic, and they contribute greatly to pulmonary disease in smokers.^2^ Numerous factors reportedly influence CC emissions from ENDS, including device power and design,^1,3−6^ liquid composition,^3,5^ and puff topography.^7,8^ Another factor that likely influences CC emissions is manufacturing variability across otherwise nominally identical ENDS products.

We have previously reported large variations in CC emissions when different ENDS atomizer heads of the same make and model were operated using the same puffing parameters, electrical power, and liquid composition and volume.^9^ Other groups have also reported large variations in CC emissions for the same ENDS product,^1,10^ and some have reported removing outliers that may be a result of “irregularities in the coil build.”^11^ One source of variability within product may derive from a varying degree of contact between the heating coil and liquid, e.g., because of an air gap between the coil and the wick.^12^ Where contact is poor, the coil can run dry, which causes the temperature to spike, and with it the rate of conversion of PG/VG to CCs.

When a powered ENDS heating coil is immersed in liquid, the coil cannot exceed the liquid boiling temperature unless it is driven at a power greater than the critical heat flux.^13^ However, if a small portion of the coil runs dry while it is powered, the local surface temperature will rise substantially above boiling to balance the received energy input,^13^ even when the coil is driven at a modest power (i.e., well below the critical flux). As the temperature rises, the reaction rates governing CC formation from PG and VG will rise exponentially.^14^ The net CC yields over several puffs will then exceed those of another nominally identical device for which the entire coil intimately contacts the wick and remains wetted during every puff. Importantly, both devices will exhibit approximately the same gross particulate matter yield, which is little affected from variations in local temperature.

In a recent study, we used nominally identical heating coils driven by a precisely regulated laboratory power supply to study the influence of various electronic cigarette liquid additives on toxicant emissions.^15^ We found large within-condition variability in toxicant emissions made insignificant any across-condition variations, except for one involving CBD oil, for which ROS emissions were significantly elevated.^15^ In this study, we re-examined the data of El-Hellani et al.^15^ by extracting and inverting computer-recorded instantaneous heating coil resistance during the individual smoking machine sessions to determine the mean peak temperature rise attained in the coil during each machine puffing session. We then examined whether the variability in computed temperature rise within condition could account for the observed variation in CC emissions.

Coil operating temperature rise during puffing was determined from the change in heating coil resistance using a well-established physical principle relating temperature of a metal conductor to its electrical resistivity. For each of the machine puffing sessions examined from El-Hellani et al.,^15^ we retrieved the instantaneous resistance (recorded every 50 ms) from the EScribe log and sorted the data from high to low. We computed the arithmetic mean of the resistance values found in the top 90th (*R*_90_) and bottom 10th (*R*_10_) percentiles. The mean peak increase in temperature during a puff, Δ*T*_max_, was then computed on the basis of the product of the relative increase in resistance and the temperature coefficient of resistance, α, of nichrome (0.0004 °C^-1^; Giancoli^16^):
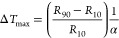


In addition to examining the resistance logs from the puffing sessions generated during the study of El-Hellani et al.,^15^ we repeated the puffing sessions using the same procedure and equipment as in the original study and rerecorded the resistance data for 10 coils that were used in the original study to determine whether those that exhibited above or below average temperature rises in the original study would exhibit the same behavior several weeks later.

The procedures used in El-Hellani et al.^15^ are briefly described for reference. Aerosols were generated using the American University of Beirut Aerosol Lab Vaping Instrument (ALVIN) connected to a Kanger Subox Mini-C ENDS device. The Subox tank was fitted with a coil head from the same manufacturer (SSOC nichrome 0.5 Ω) and powered using a regulated DC power supply at 30 W. The manufacturer specifications for the Subox device indicate a maximum rated operating power of 50 W. The power supply was controlled by a DNA200 circuit board (2018 Evolv LLC). Data including voltage, power, current, and resistance were recorded in 50 ms intervals using EScribe Suite (2018 Evolv LLC) (Figure S1). Each tank was filled with a 30/70 (v/v) mixture of analytical grade PG (≥99.5%, CAS 57-55-6) and VG (99.0–101.0%, CAS 56-81-50) purchased from Sigma-Aldrich. Analytical grade PG (≥99.5%, CAS 57-55-6) and VG (99.0–101.0%, CAS 56-81-5) liquids were procured from Sigma-Aldrich Corporation and used to prepare a solution with a 30/70 PG/VG ratio. Each aerosol sample was generated using a brand-new coil head. Puff topography was held constant at 10 puffs of 4 s duration, 10 s interpuff interval, and 8 liters per minute (LPM) flow rate, thereby approximating the average flow rate and duration obtained in a clinical setting using the same device as reported by Hiler et al.^17^ Finally, we preconditioned each ENDS by filling the tank with liquid, thereby allowing the device to sit in the vertical position for at least 30 min and then drawing 3 puffs at 15 W; the filter pads were replaced following the preconditioning step. Throughout all sessions, we maintained the liquid level in the ENDS above the wicking holes.

For each sampling session, the aerosol exiting the mouth end of the ENDS was drawn through a Gelman type A/E 47 mm glass fiber filter pad followed by a 2,4-dinitrophenylhydrazine (DNPH) cartridge. Total particulate matter (TPM) was determined by weighing the filter assembly before and after each session. CCs were quantified by extracting the DNPH cartridges in 90/10 (v/v) ethanol/acetonitrile and analyzing the prepared extract by high-performance liquid chromatography with ultraviolet detection (HPLC-UV), as described in Al Rashidi et al.^18^ The species analyzed and the limit of detection and limit of quantitation were, respectively, as follows (μg): formaldehyde, 0.006 and 0.019; acetaldehyde, 0.004 and 0.012; acetone, 0.002 and 0.006; acrolein, 0.002 and 0.006; propionaldehyde, 0.004 and 0.014; benzaldehyde 0.004 and 0.013; valeraldehyde, 0.002 and 0.006; hexaldehyde, 0.002 and 0.006; glyoxal, 0.005 and 0.018; and methyl glyoxal, 0.002 and 0.008. To remove extraneous variables from the analysis, we excluded from the original data set of El-Hellani et al.^15^ seven samples for which the resistance data were missing, nine samples which were generated at higher power (45 W vs 30 W), and 35 samples which were generated either using CBD oil or liquids with other than 30/70 PG/VG ratios.

We found that computed Δ*T*_max_ varied between 112 and 489 °C across samples. Emissions of CC species varied over 2 orders of magnitude, while total particulate matter emissions across devices were relatively consistent (RSD < 10%). A summary of the results is presented in Table 1.

**Table 1:** Total Particulate Matter, Maximum Temperature Rise, and Total Carbonyl Compounds in 15 Puffs Across 75 Repeated Measurements, Each with a New Kanger SSOC Atomizer

	average (SD)	median	range	RSD (%)
TPM (mg)	579(36.4)	584	454–645	6.29
Δ*T*_max_ (°C)	312(77.7)	315	112–589	25
carbonyls (μg)				
formaldehyde	19.1(83.3)	3.04	0.219–636	437
acetaldehyde	122(79.5)	106	66.9–605	65.1
acetone	142(40.5)	120	97.4–218	28.6
acrolein	3.67(13.3)	2.11	0–97.6	363
propionaldehyde	4(9.06)	2.44	0–62.2	226
crotonaldehyde	10.5(5.5)	9.19	5.17–40	52.2
methacrolein	27.1(6.44)	23.9	19.5–39.5	23.7
butyraldehyde	14(9.53)	16.6	0–31.3	67.9
valeraldehyde	0.0867(0.751)	0	0–6.5	866
hexaldehyde	2.15(2.31)	2.42	0–8.44	107
glyoxal	19.8(20.4)	16.7	9.42–190	103
methyl glyoxal	90(118)	58	24.8–698	131
sum of carbonyls	450(313)	393	47.7–2260	69.6

We also found that carbonyl emissions increased significantly with temperature (formaldehyde, *R*2 = 0.37, *p* < 0.0001; CCs, *R*2 = 0.12, *p* < 0.001; exponential models) (Figure 1 and Figure S2). As shown in Figure 1, for atomizer coils whose Δ*T*_max_ remained below 300 °C, the formaldehyde yields never exceeded 10 μg. For coils whose Δ*T*_max_ exceeded 300 °C, the formaldehyde yields could exceed 500 μg. The attainment of a Δ*T*_max_ of 300 °C may be a proxy indicator for the coil attaining film boiling for a significant portion of the total puffing time. As we have previously shown, formaldehyde and other CC yields rise greatly when the coil reaches film boiling.^13^

**Figure 1:**
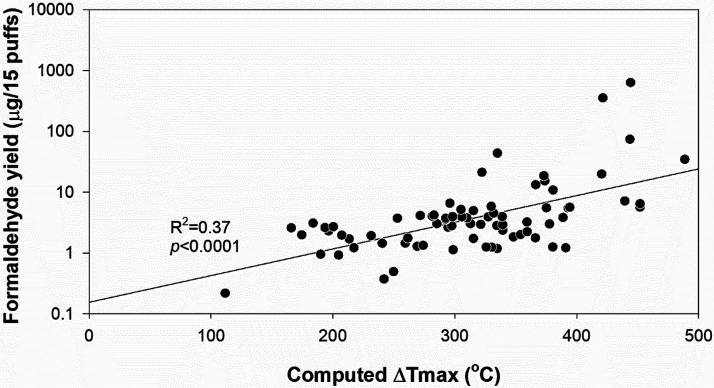
Formaldehyde yields vs Δ*T*_max_ (*N* = 75); log scale. An exponential model was used to fit formaldehyde yield as a function of Δ*T*_max_.

For the coils that were tested a second time for the current study, we found that four of the five coils that initially exhibited a Δ*T*_max_ exceeding 300 °C on the first trial exceeded 300 °C on the second trial. Of the five that exhibited a Δ*T*_max_ below 300 °C on the first trial, four remained below 300 °C on the second trial (Figure S3).

Because carbonyl emissions from ENDSs under controlled conditions have shown much greater variability than exhibited with other species of interest, such as nicotine or total particulate matter, we examined systematically the hypothesis that this variability may be driven by fluctuations in operating temperature across nominally identical devices. This hypothesis was informed by the fact that unlike nicotine, CC emissions derive from thermal degradation of the liquid during a puff. We found that temperature rise varied widely across devices, spanning a 375 °C range, and that CC emissions varied by up to two orders of magnitude, in which a greater Δ*T*_max_ resulted in exponentially higher CC and carcinogenic formaldehyde emissions. In all cases where formaldehyde emissions were greater than 20 μg, which is the reported yield of 3R4F cigarettes,^19^ the computed Δ*T*_max_ was above 300 °C. We also found that the distribution of CC emissions across devices was highly skewed. As a result, a few devices accounted for most of the CC toxicant yields summed across the 75 devices. For example, nine devices accounted for more than 85% of the ensemble total formaldehyde emissions (Figure 2). At least for the Subox Mini C, this finding suggests that a significant reduction in population-wide toxicant exposure could be realized if the manufacturer were required to employ tighter manufacturing tolerances or a temperature control algorithm in the power unit.

**Figure 2:**
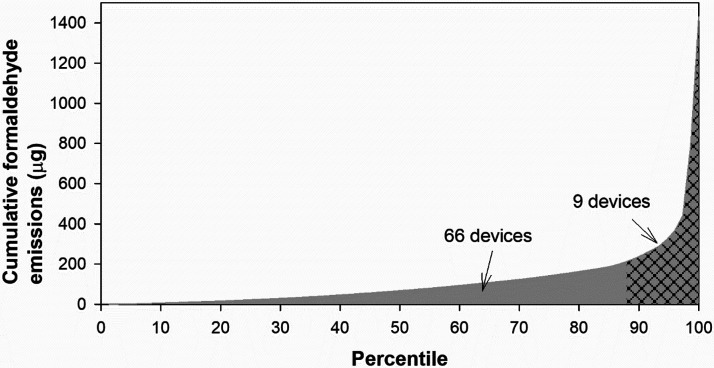
Cumulative formaldehyde emissions versus percentile of samples.

When a subset of high/low temperature operation devices were tested several weeks after the initial study, most of those that exhibited high temperature during the initial study also exhibited high-temperature operation with the repeated measurements. Similarly, most of those that exhibited low temperatures in the original study also did so in the repeated measurements. These findings suggest that different operating temperature regimes are intrinsic to the particular device and support the notion that manufacturing variability may drive pulmonary toxicant emissions in some ENDSs.

In conclusion, we found that CC emissions vary widely across nominally identical ENDS products operated at the same power and that this variability is associated with temperature fluctuations that likely stem from manufacturing variations. Our findings reinforce the notion that ENDS product performance metrics must be considered alongside design-based regulations (e.g., limits on liquid composition or power) to effectively protect public health.

## Supporting Information Available

Additional figures, including a schematic of the experimental setup and additional results: total CCs vs Δ*T*_max_ and original and repeated measures of Δ*T*_max_ (PDF)

## Abbreviations

ALVIN: American University of Beirut Aerosol Lab Vaping Instrument
CCs: carbonyl compounds
DNPH: 2,4-dinitrophenylhydrazine
ENDS: electronic nicotine delivery system
FDA: Food and Drug Administration
HPLC-UV: high-performance liquid chromatography with ultraviolet
NIH: National Institute of Health
PG: propylene glycol
*R*: resistance
RSD: relative standard deviation
SD: standard deviation
TCR: temperature coefficient of resistance
TPM: total particulate matter
VG: vegetable glycerin
