# Supplementary material for: Carbonyl Emissions and Heating Temperatures across 75 Nominally Identical Electronic Nicotine Delivery System Products: Do Manufacturing Variations Drive Pulmonary Toxicant Exposure?
Source: Chem Res Toxicol. 2023 Feb 16;36(3):342–6. doi: 10.1021/acs.chemrestox.2c00391 (PMC10031554; doi:10.1021/acs.chemrestox.2c00391)
Supplement: Supplementary file 1 — tx2c00391_si_001.pdf [file tx2c00391_si_001.pdf]

Supplementary Information for

Carbonyl emissions and heating temperatures across seventy-five nominally identical ENDS products: do manufacturing variations drive pulmonary toxicant exposure?

Soha Talih, PhD[†,‡], Rola Salman, BS[†,‡], Nareg Karaoghlanian, BE[†,‡], Ahmad El-Hellani, PhD[§], Alan Shihadeh, ScD[†,‡]*

[†] Mechanical Engineering Department, Maroun Semaan Faculty of Engineering and Architecture, American University of Beirut, Bliss Street, PO. Box 11-0236, Beirut, Lebanon

[‡] Center for the Study of Tobacco Products, Virginia Commonwealth University, 821 West Franklin Street, Richmond, Virginia 23284, United States

[§] Environmental Health Sciences, College of Public Health, The Ohio State University, 1841 Neil Ave., Columbus, OH 43210, United States

* Corresponding Author: Alan Shihadeh
Tel: + 961 1 344444
Email address: as20@aub.edu.lb
Address: American University of Beirut
PO Box 11-0236 Beirut, Lebanon

Table of Contents:

Figure S1 Schematic of the experimental setup. The ENDS is controlled by a DNA200 circuit board (© 2018 Evolv LLC). EScribe Suite (© 2018 Evolv LLC) was used to collect data. The left figure shows an example of the resistance measurements collected from the EScribe Suite from one puffing session (puffs: 1-10) using a single ENDS. The arithmetic mean of the resistance values found in the top 90th (R90) and bottom 10th (R10) percentiles were then computed. The mean peak increase in temperature during a puff, ΔT_{max}, was then computed based on the product of the relative increase in resistance and the temperature coefficient of resistance α, such as $\Delta T_{max} = \left(\frac{R_{90} - R_{10}}{R_{10}}\right)\frac{1}{\alpha}$.

Figure S2 Total CCs vs. ΔT_{max} (N=75). An exponential model was used to fit total CCs as a function of ΔT_{max} resulting in an R^2=0.14, p<0.01.

Figure S3 Original and repeated measures of ΔTmax (N=10 devices). Four of the five coils that initially exhibited a ΔT_{max} exceeding 300°C (dashed line) on the original trial exceeded 300°C on the repeated trial. Of the five that exhibited a ΔT_{max} below 300°C on the original trial, four remained below 300°C on the repeated trial.

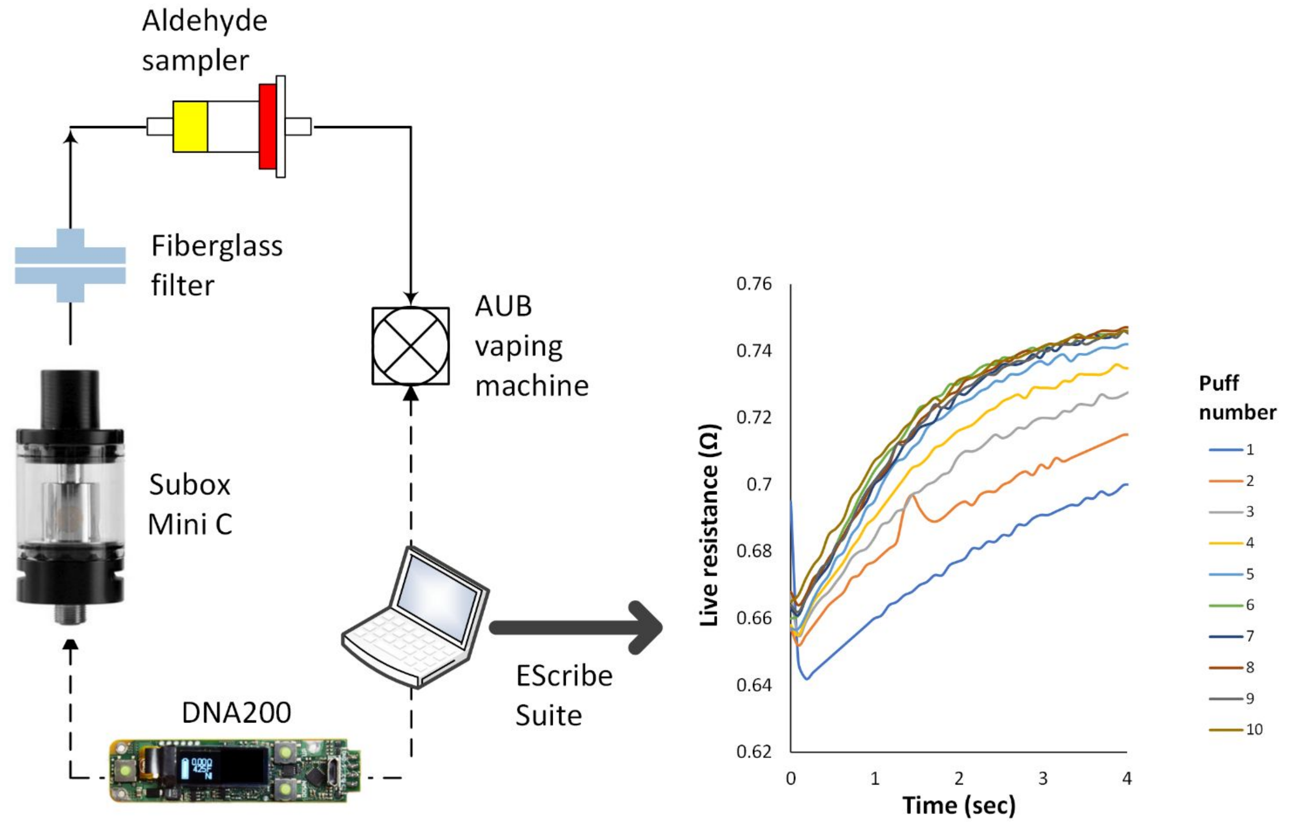

Figure S1 Schematic of the experimental setup. The ENDS is controlled by a DNA200 circuit board (© 2018 Evolv LLC). EScribe Suite (© 2018 Evolv LLC) was used to collect data. The left figure shows an example of the resistance measurements collected from the EScribe Suite from one puffing session (puffs: 1-10) using a single ENDS. The arithmetic mean of the resistance values found in the top 90th (R_{90}) and bottom 10th (R_{10}) percentiles were then computed. The mean peak increase in temperature during a puff, ΔT_{max}, was then computed based on the product of the relative increase in resistance and the temperature coefficient of resistance α, such as $\Delta T_{max} = \left(\frac{R_{90} - R_{10}}{R_{10}}\right)\frac{1}{\alpha}$.

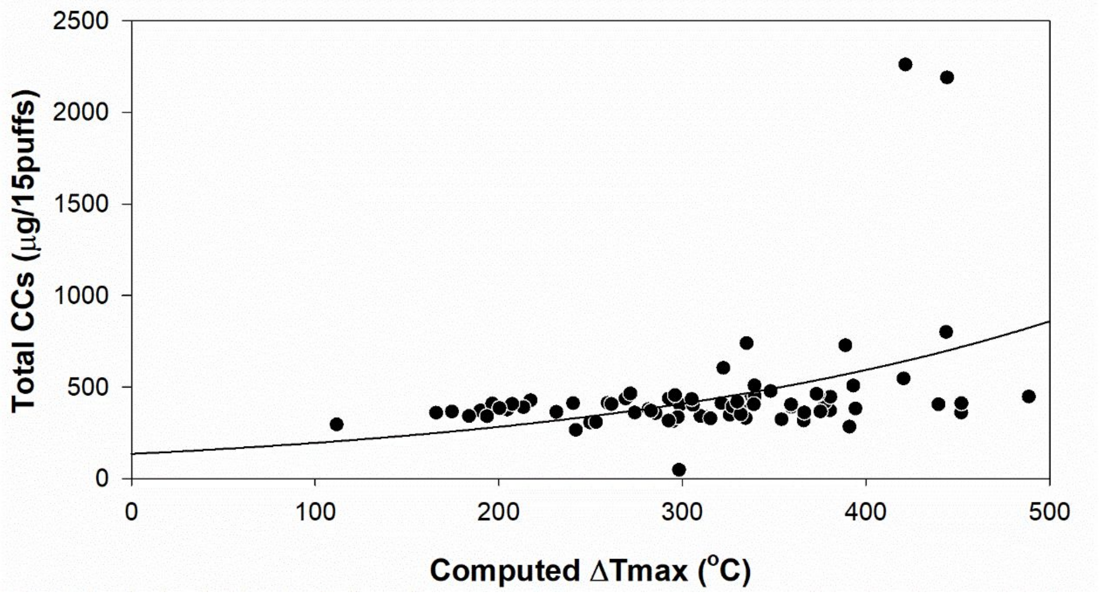

Figure S2 Total CCs vs. ΔT_{max} (N=75). An exponential model was used to fit total CCs as a function of ΔT_{max} resulting in an R^2=0.14, p<0.01.

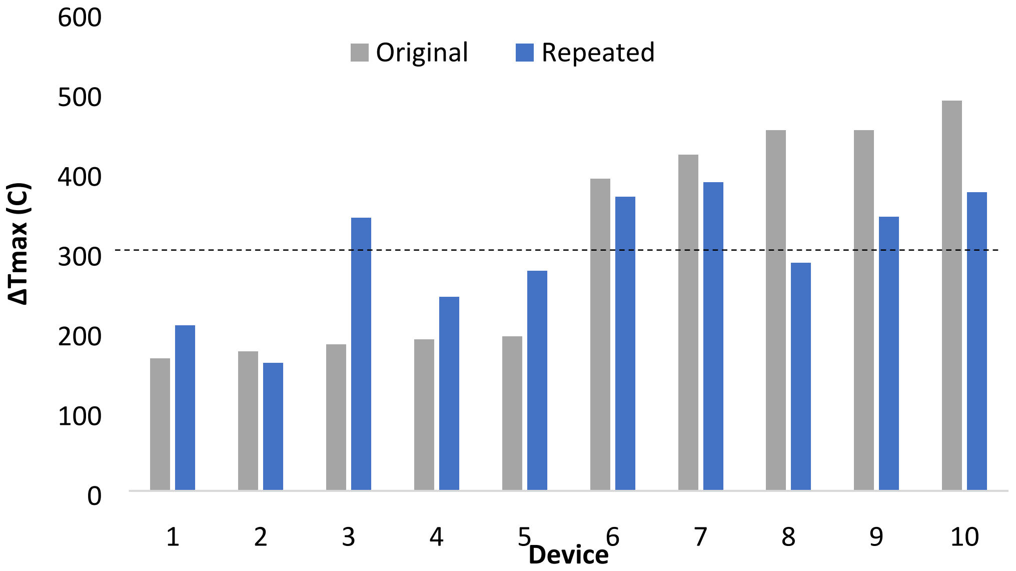

Figure S3 Original and repeated measures of ΔTmax (N=10 devices). Four of the five coils that initially exhibited a ΔT_{max} exceeding 300°C (dashed line) on the original trial exceeded 300°C on the repeated trial. Of the five that exhibited a ΔT_{max} below 300°C on the original trial, four remained below 300°C on the repeated trial.
